# Regulation of Energy Metabolism during Early B Lymphocyte Development

**DOI:** 10.3390/ijms19082192

**Published:** 2018-07-27

**Authors:** Sophia Urbanczyk, Merle Stein, Wolfgang Schuh, Hans-Martin Jäck, Dimitrios Mougiakakos, Dirk Mielenz

**Affiliations:** 1 Division of Molecular Immunology, Nikolaus-Fiebiger-Center, Friedrich-Alexander-Universität Erlangen-Nürnberg (FAU), 91054 Erlangen, Germany; sophia.urbanczyk@uk-erlangen.de (S.U.); wolfgang.schuh@uk-erlangen.de (W.S.); hjaeck@gmail.com (H.-M.J.); 2 Institute of Comparative Molecular Endocrinology (CME), University of Ulm, 89081 Ulm, Germany; merle.stein@uni-ulm.de; 3 Department of Internal Medicine V, University Hospital, Friedrich-Alexander-Universität Erlangen-Nürnberg (FAU), 91054 Erlangen, Germany; dimitrios.mougiakakos@uk-erlangen.de

**Keywords:** B lymphocyte development, metabolism, EFhd1, pre-BCR, mitochondria, mitoflash, oxidative phosphorylation, glycolysis

## Abstract

The most important feature of humoral immunity is the adaptation of the diversity of newly generated B cell receptors, that is, the antigen receptor repertoire, to the body’s own and foreign structures. This includes the transient propagation of B progenitor cells and B cells, which possess receptors that are positively selected via anabolic signalling pathways under highly competitive conditions. The metabolic regulation of early B-cell development thus has important consequences for the expansion of normal or malignant pre-B cell clones. In addition, cellular senescence programs based on the expression of B cell identity factors, such as Pax5, act to prevent excessive proliferation and cellular deviation. Here, we review the basic mechanisms underlying the regulation of glycolysis and oxidative phosphorylation during early B cell development in bone marrow. We focus on the regulation of glycolysis and mitochondrial oxidative phosphorylation at the transition from non-transformed pro- to pre-B cells and discuss some ongoing issues. We introduce Swiprosin-2/EFhd1 as a potential regulator of glycolysis in pro-B cells that has also been linked to Ca^2+^-mediated mitoflashes. Mitoflashes are bioenergetic mitochondrial events that control mitochondrial metabolism and signalling in both healthy and disease states. We discuss how Ca^2+^ fluctuations in pro- and pre-B cells may translate into mitoflashes in early B cells and speculate about the consequences of these changes.

## 1. B Lymphocyte Development

**Figure 1:**
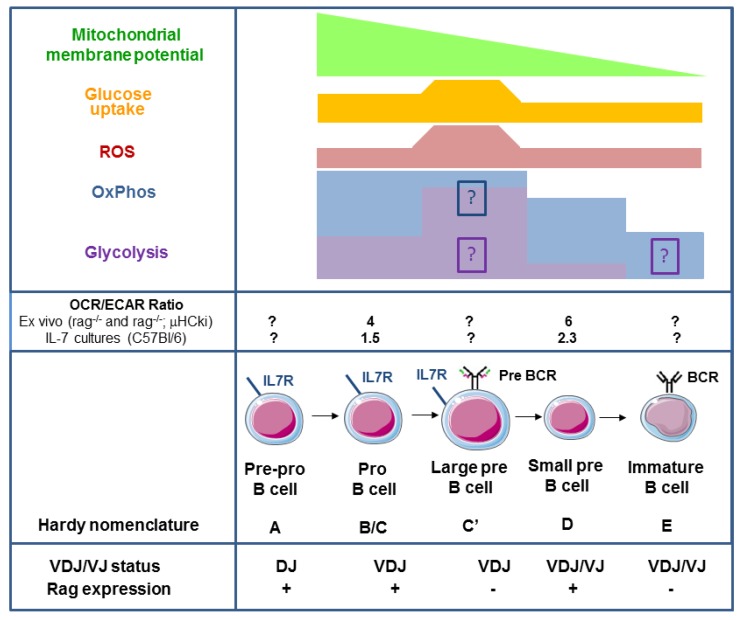
Relationship between oxidative phosphorylation and glycolysis during early murine B cell development. Summary of experimental determinations of the mitochondrial membrane potential Δψ_m_, glucose uptake, ROS production, Oxphos and glycolysis during B cell development from pro- to large and from small pre-B to immature B cells [21,22]. Extracellular flux analysis of pro- and small pre-B cells (mainly small) obtained from rag2^−/−^ or rag2^−/−^; μHC knock-in mice or from sorted wildtype pro- and pre-B cells (mainly small) obtained from IL-7 cultures revealed a decline in both glycolysis and OxPhos, with the decline in glycolysis being more pronounced [21]. Consequently, small pre-B cells show a higher OxPhos to glycolysis ratio in both systems, with a lower ratio observed in the IL-7 cultures. Immature B cells reveal an even lower rate of OxPhos [22]. Missing data are indicated by question marks. The increase in glycolysis and OxPhos in large pre-B cells is speculative (see boxed question marks in matching colours) and based on literature reviews.

B lymphocytes develop in adult vertebrates in the bone marrow (BM). They are derived from pluripotent stem cells and develop through the following stages: hematopoietic stem cells (HSCs); common lymphoid progenitors (CLPs); B cell-biased lymphoid progenitors (BLPs); and pre-pro-, pro- and pre-B cells. B cell precursors require cell contact and specific niches in the BM for their survival and growth [1]. Proliferative HSC and pre-pro-B cells, the earliest committed B lymphocyte progenitors, develop in the vicinity of sinusoids [2,3,4]. Pre-pro-B cells localize next to CXCL12 (also: SDF-1, stromal cell derived factor)-abundant reticular (CAR) cells, whereas pro-B cells are found adjacent to IL-7-expressing stromal cells, the majority of which are in close contact with the vasculature [5]. Pre-B cells localize near Galectin-1-expressing cells [1]. Each of these different niches possesses different oxygen tensions, indicating that there is a need to adapt mitochondrial respiration during different B cell developmental stages [6]. The specific characteristics of the niches required for early B lymphocyte development need further exploration and are at least partially and indirectly dependent on osteoblasts [2,4]. The active migration of cells towards their respective niches is induced by chemokines such as CXCL12 [3,5]. CXCL12 and CXCR4, the only receptor for CXCL12, are required for HSC and B cell development in a non-redundant manner [1,7,8,9], with CXCL12 eliciting an intracellular Ca^2+^ signal [7]. The first step of B lymphocyte development is controlled by the transcription factors (TFs) PU.1 and Ikaros (IKZF1), both of which are expressed in CLPs. Progenitors then commit to the B cell lineage by expressing E2A, EBF-1 and Pax-5 (reviewed in [10]). Pre-pro-B cells develop into pro-B cells (Figure 1), in which proliferation is supported by numerous factors, especially the cytokine interleukin 7 (IL-7) as well as CXCL12 and stem cell factor (SCF) [1]. Another important factor supporting early B cell development is Fms-like tyrosine kinase (Flt) 3 ligand [1]. B lymphocyte development follows defined stages that can be distinguished by the expression of cell surface markers, genetic rearrangements of Immunoglobulin (Ig) heavy and light chain loci and cell size and mitotic activity [11] (Figure 1; for detailed reviews see [1,12,13,14]). 

In pro-B cells, binding of IL-7 to the IL-7 receptor drives the expression of the anti-apoptotic molecules Bcl-2 and myeloid-cell leukaemia sequence 1 (MCL1), enhancing survival and proliferation [3,15]. In vitro IL-7 induces proliferation in pro-B cells (Hardy fraction B and C) but not in further differentiated B cells [11]. This IL-7 dependency appears to be stronger in mice than in humans [16]. During development from pro-B cells to immature B cells, IL-7R is downregulated and responsiveness to IL-7 decreases [17,18]. A much smaller proportion of pre-B cells and immature B cells is found with higher concentrations of IL-7 but this is not due to the active suppression of differentiation. In fact, pro-B cells can also differentiate into pre-B cells and sIgM^+^ cells in the presence of higher IL-7 concentrations but these cells are outnumbered by proliferating pro-B cells [18]. The expression of Rag1 and 2 by pro-B cells allows diverse to joining (D-J) and variable D-J (VDJ) recombination of the gene segments that encode the μ heavy chain (μHC) of the B cell receptor (BCR) in pre-pro-B cells (Fraction A; Hardy et al. [11]) and pro-B cells (Hardy fraction B/C), respectively (Figure 1; reviewed in detail in [12,13,14]). After productive VDJ recombination, the newly formed μHC can pair with the surrogate light chain complex consisting of VpreB and λ5, resulting in pre-BCR expression and the appearance of large pre-B cells (Hardy fraction C/C’) (Figure 1). Mice deficient in either of these Rag genes show a developmental B lymphocyte block and accumulate pro-B cells in the BM because rearrangement of µHC D-J and then VDJ elements cannot take place [19,20]. 

Ectopic expression of the µHC on a Rag2^−/−^ background in mice led to the development of phenotypic pre-B cells, while the introduction of µHC and lambda (λ)-LC led to the production of peripheral, monoclonal and immunoglobulin-secreting B cells [20]. The Pre-BCR elicits an increase in the cytosolic Ca^2+^ concentration [23,24,25,26] and acts as an inducible proliferative signal in pre-B cells with an expansion factor of 20–100 (approximately 4–6 cell divisions) [27]. Hence, B cell clones with an optimal pre-BCR signalling strength, based on μHC idiotype, will expand (pre-BCR signal 1) [28]. This defines the basis for the (mostly autoreactive) pre-immune BCR repertoire, which represents a direct link between metabolism, growth control and autoreactivity [29,30]. The mechanisms by which the pre-BCR induces this expansion signal at the structural level have been reviewed elsewhere [5,31,32]. Expression of the pre-BCR also inhibits further rearrangements of the V to DJ loci in the not yet re-arranged µHC allele (allelic exclusion) [28]. After the first round of clonal expansion, pre-B cells become quiescent again and decrease in size (pre-BCR signal 2). In these resting, small pre-B cells (Hardy fraction D), gene rearrangements occur in the V and J segments encoding the BCR light chain [14]. Successful VJ rearrangement gives rise to light chain protein, BCR expression and naïve, immature B cells (Hardy fraction E) (Figure 1). Immature B cells then complete development into resting mature follicular and marginal zone B cells in the spleen [33]. As outlined above, pro-B cells proliferate in response to IL-7, expand transiently into large pre-B cells upon early pre-BCR expression and then become quiescent as small pre-B cells again to allow VJ recombination to occur (reviewed in Clark et al. [14]) (Figure 1). The proximal signalling pathways that control these transitions have been reviewed in detail elsewhere [14,32,34] but several questions remain incompletely answered. For example, how is pro-B cell proliferation maintained homeostatically? How does IL-7 affect early B cell metabolism? How do pre-BCRs, IL-7 and nutrients control the transient expansion of large pre-B cells and the subsequent quiescence of small pre-B cells? The purpose of this review is to summarize what is currently known about metabolism during early B cell development. 

## 2. Oxidative Phosphorylation and Glycolysis in Pro- and Pre-B Cells

The ultimate downstream biochemical events that supply cells with adenosine triphosphate (ATP) are glycolysis and mitochondrial oxidative phosphorylation (OxPhos) (reviewed in detail in the context of lymphocytes [35]). The oxidation of fatty acids (FAs), carbohydrates and amino acids is coupled to ATP synthesis in mitochondria by the proton gradient across the inner mitochondrial membrane (IMM). The proton gradient (∆pH_m_) across the IMM is established by the electron transport chain (ETC) by mitochondrial respiratory chain complexes I, III and IV, which pump protons from the matrix into the mitochondrial intermembrane space [35]. ∆pH and the mitochondrial membrane potential (∆ψm) contribute independently to the proton motive force (∆*p*) that drives the synthesis of ATP via the ATP synthase complex (complex V) (∆*p* = ∆pH_m_ + ∆ψm) [36]. The concentration of ATP relative to that of ADP and AMP is an indicator of the cellular energy status and is sensed by a kinase complex called adenosine monophosphate – activated protein kinase (AMPK). When the AMP/ATP ratio reaches a certain threshold, AMPK becomes activated to support catabolic pathways and ensure an ongoing energy supply. AMPK activity promotes mitochondrial biogenesis and autophagy and represses the mammalian target of Rapamycin (mTOR) pathway [37,38,39]. 

**Figure 2:**
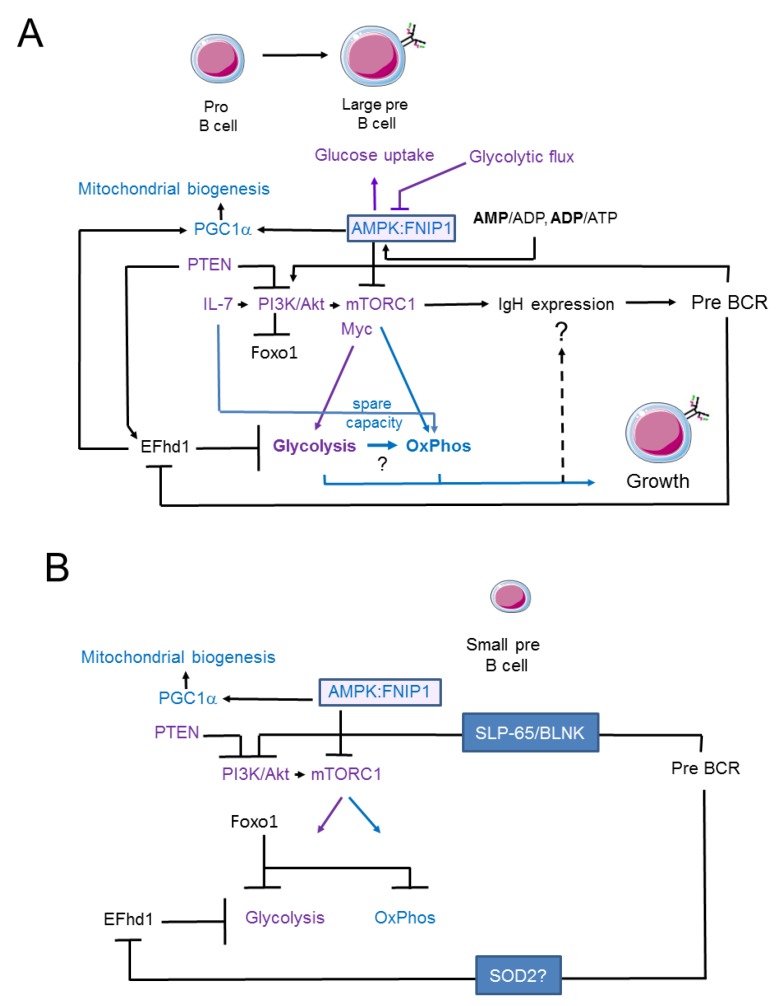
Relationship between oxidative phosphorylation and glycolysis during early murine B cell development. In pro- and large pre-B cells, growth is controlled by IL-7 and the pre-BCR, which lead to the activation of the PI3K/Akt/mTOR pathway. mTORC1 activates glycolysis and OxPhos and is required for the protein expression of μHC, which controls pre-BCR expression and signalling. Whether control of μHC protein expression occurs via glycolysis and/or OxPhos is not clear. Surface expression of the pre-BCR leads to the downregulation of EFhd1, a Ca^2+^-binding protein localized on the inner mitochondrial membrane. EFhd1 suppresses glycolysis in transformed pro-B cells and is induced by PTEN. In primary pre-B cells, overexpression of EFhd1 induces PGC1α, which is also controlled by AMPK1, a negative regulator of anabolic pathways and a positive regulator of catabolic pathways. AMPK activity is controlled by ADP/AMP and ATP/ADP ratios and fructose 1,6 bisphosphate and is an indicator of glycolytic flux. AMPK activity is also modified by FNIP1. Loss of FNIP1 by genetic ablation leads to anabolic exhaustion in pro-/pre-B cells. IL-7 increases the mitochondrial spare capacity in pre-B cells. (**A**) The network in pro- and large pre-B cells with an activated PI3K pathway and inactivated Foxo1; and (**B**) the network in small pre-B cells, in which the PI3K and mTORC1 pathways are inactivated but Foxo1 is activated. It is unclear whether large pre-B cells depend solely on glycolysis or whether glycolytic pathways are mutually linked to OxPhos.

Inhibition experiments performed with 2-deoxyglucose (2-DG), a non-hydrolysable glucose analogue that blocks glycolysis, have shown that pro-/early/pre-B cells depend on the glycolytic pathway, whereas late (small) pre-B cells do not [40]. In contrast, a lack of glucose did not prevent the development of IgM-positive cells in vitro in total BM cultures [41]. It should be noted that 2-DG has off-target effects, including endoplasmic reticulum (ER) stress, autophagy induction, interference with mannose and reduced protein *N*-glycosylation (reviewed in detail in [42]). Hence, these findings need to be reconciled. However, the experiments performed by Kojima et al. revealed the existence of a metabolic checkpoint in early B cell development. This finding was corroborated by a genetic screen that revealed the existence of a metabolic checkpoint controlled by folliculin interacting protein 1 (Fnip1). Fnip1 forms a complex with AMPK [39] and in Fnip1^−/−^ mice, B cell development is blocked at the large pre-B cell stage due to an imbalance in metabolism [41]. In WT BM B cell cultures derived from total BM cells grown in the presence of IL-7, SCF and Flt 3 ligand for 48 h, depleting the cells of glucose, glutamine or essential amino acids did not affect the number of developing IgM-positive B cells. However, Fnip1^−/−^ B cell progenitors were sensitive to these depletions, indicating a state of energy exhaustion. Under the same experimental conditions, oligomycin (an inhibitor of ATP synthase activity in mitochondrial respiratory chain complex V) at 10 or 50 nM did not affect the appearance of-IgM positive WT B cells, while 10 nM oligomycin did alter the appearance of these cells in Fnip1^−/−^ cultures. Extracellular flux analyses performed with a Seahorse analyser showed that pro-/pre-B cells responded to IL-7 by increasing their oxygen consumption rate (OCR; an indicator of oxidative phosphorylation/OxPhos) (We would like to add, as a technical note not related specifically to the cited publication [41], that measuring OCR in a Seahorse analyser does not provide information about the substrates fuelling the TCA cycle. Information about these substrates can be obtained by using labelled substrates or inhibitors. For instance, FA importation into mitochondria can be inhibited by Etomoxir but at high concentrations, Etomoxir exerts off-target effects, including inhibiting complex I of the electron transport chain [43]) and extracellular acidification rate (ECAR; an indicator of glycolysis) (ECAR measured in a Seahorse analyser represents a pH measurement. To ensure that an observed decrease in extracellular pH is due to an increase in lactate secretion that occurs as a consequence of glycolysis, it is recommended that lactate should be measured or ^13^C-labeled glucose be tracked) [41]. However, Fnip1^−/−^ pro/pre-B cells responded better. To determine which substrates fuel the observed increase in OCR, sorted pro-/pre-B cells were treated with 2-DG and Etomoxir. Both treatments reduced the OCR in WT cells but it was reduced even more in Fnip1^−/−^ pro-/pre-B cells. These data suggested that pro-/pre-B cells utilize glucose and FA for OxPhos and that Fnip1 renders pro-/pre-B cells resilient to inhibition of glycolysis and of FA oxidation. Further experiments connected this metabolic checkpoint to the Fnip1:AMPK complex and the pro-/pre-B cell transition (Figure 2A). The anabolic ATP exhaustion observed in Fnip1^−/−^ pro-/pre-B cells is likely mediated by an increase in *rps6ka1* expression [41]. A similar mechanism has been observed in transformed haploinsufficient Phosphatase and Tensin homologue (PTEN)^-/+^ and PTEN^−/−^ pre-B acute lymphoblastic leukaemia (ALL) cells [41,44]. While the experiments performed by Kojima et al. and Park et al. were seminal, measurements of OxPhos and glycolysis in discrete pro- and pre-B cell populations have not yet been performed under more defined conditions (e.g., medium with IL-7 only). Thus, we analysed metabolism in discrete pro- and pre-B cells (Figure 1) [21]. Mitochondrial mass relative to cell size is decreased in large pre-B cells but remains constant during later B cell development [21]. Pro-B cells exhibited the highest ∆ψμ; ∆ψμ is then significantly lower in small pre-B cells and declines further during development. Reactive oxygen species (ROS) production, as measured by 2′-7′-dichlorodihydrofluorescein diacetate (DCDFA, a dye that does not specifically quantify mitochondrial ROS) and glucose uptake are highest in large pre-B cells but reduced in small pre-B cells, supporting the data described by Kojima and colleagues [40]. To measure glycolysis and OxPhos directly in pro- and pre-B cells, we established a μHC knock-in (ki) mouse model (33.C9μHCki) and crossed these mice to Rag1^−/−^ mice [19] (Rag1^−/−^;33.C9μHCki) [21]. Pre-B cells obtained from Rag1^−/−^;33.C9μHCki mice are mainly small. Extracellular flux analyses performed with sorted primary pro- and pre-B cells obtained from this system revealed that in general, under normoxic conditions, OCR and ECAR were lower in Rag1^−/−^;33.C9μHCki pre-B cells than pro-B cells. These data were confirmed by Zeng et al., who also analysed immature B cells, which have an OCR similar to that of small pre-B cells [22]. In contrast to Zeng et al. we also assessed glycolysis. In our experiments, glycolysis (evaluated by ECAR) was significantly reduced relative to OCR in small pre- versus pro-B cells, resulting in a higher OCR/ECAR ratio (Figure 1). However, the contributing mechanisms and consequences of the alterations in OCR/ECAR ratios and mitochondrial spare capacity observed in this system require more study. Nevertheless, we noted that the OCR/ECAR ratio was in general lower in IL-7 cultures, suggesting that IL-7 promotes glycolysis (Figure 1). In fact, IL-7 promotes glycolysis by activating Akt [13,45,46] and this might be important in IL-7-rich niches in BM [1,6]. IL-7 also appears to elevate mitochondrial spare capacity, perhaps via the pyruvate that is generated by glycolysis and directed towards the tricarbon (TCA) cycle (Figure 2A). The data described in Park et al. [41] do indeed imply that mixed pro-/pre-B cell cultures use pyruvate derived from glycolysis to fuel and maintain OxPhos but more experiments are needed to define the TCA substrates used in pro- and pre-B cells. In summary, pre-BCR expression ultimately promotes metabolic quiescence (pre-BCR signal 2) by reducing glycolysis (as defined by ECAR using a Seahorse analyser), resulting in an increased OCR/ECAR ratio. The decrease in glycolysis observed in small pre-B cells compared to pro-B cells is in agreement with the proposal that Akt is inactivated [32,47] (pre-BCR signal 2) and that glucose up-take [21] and responsiveness to IL-7 are reduced.

It appears that the reduction of metabolism observed in small pre-B cells is maintained in immature B cells [22]. Additionally, resting splenic B cells exhibit low metabolic activity [48] and consume FA to produce ATP via OxPhos, as shown by metabolic tracking of FA [48]. Upon BCR activation or by lipopolysaccharide (LPS) mediated TLR4 activation, normal but not anergic splenic murine B cells again upregulated both OxPhos and glycolysis in a Myc-dependent manner in a balanced ratio [48,49,50], concomitant with an increase in the glucose transporter *glut1*. The newly activated B cells then oxidize glutamine and pyruvate [48]. Interestingly, murine peritoneal B1 B cells are metabolically more active than follicular B cells and depend on glycolysis [51]. Glycolysis (analysed in inhibition experiments performed with dichloroacetate, an inhibitor of pyruvate dehydrogenase) also supported the secretion of antibodies from murine and human B cells both in vitro and in vivo [48]. Glucose taken up by plasma cells is mainly used for antibody glycosylation [52]. No information is available regarding differences between follicular and marginal zone B cells or whether small pre-B cells or immature B cells rely on FA. In addition, extracellular flux analyses of primary pro-and small pre-B cells failed to identify large pre-B cells for technical reasons. Large pre-B cells could, in the future, potentially be enriched from Irf4/8 double [53] or BLNK/SLP-65 knock-out mice [54], which fail to downregulate pre-BCR, show hyperproliferation of large pre-B cells and are prone to malignant transformation. It is likely that glycolysis is increased in large pre-B cells and this would provide more energy and more anaplerotic reactions for macromolecules and intermediate products, thereby protecting cells from ROS [55], in addition to more pyruvate to support mitochondrial ATP production (Figure 1). A genetic in vivo system that allows the mitochondrial respiratory chain to be manipulated could address this question. For instance, experiments performed in mice with an inducible deletion of the mitochondrial pyruvate importer Mcp2 (in *Mcp2^fl/fl^;ROSA26 CreER* mice) revealed that plasma cells in the BM rely on this mechanism [52]. 

A caveat of all the studies mentioned so far is that ex vivo experiments are generally performed under normoxic conditions, while many parts of the BM and several of its niches are hypoxic [6]. Immune cells adapt to hypoxia by stabilizing HIF at the protein level [56]. Indeed, HIF1α deficiency impaired early B cell development in Rag2^−/−^ blastocyst complementation chimeras by reducing the number of proliferating CD43^-^HSA^+^B220^+^ cells [57]. On the other hand, B cell development in the BM of HIF1α^fl/fl^ or HIF2α^fl/fl^ mice crossed to Mb1-Cre mice is normal [58]. Although the CD43^-^HSA^+^B220^+^ cells affected by HIF1α deficiency are likely proliferating pre B cells, there is the possibility that HIF1α mediated metabolic adaptations influence B cell development already before the mb-1 promotor is active. HIF-1α controls glycolysis in BM precursor B cells in a developmental stage-specific manner by regulating the genes that encode glucose transporters and the key glycolytic enzyme 6-phosphofructo-2-kinase/fructose-2,6-bishosphatase 3 [40]. Interestingly, HIF1α-deficient B cell progenitors compensated for defects in glycolytic enzymes by increasing the expression of respiratory chain-related genes and TCA-related genes, enabling more efficient pyruvate usage [40]. These data reveal that B cell progenitors are metabolically flexible and show a propensity to adapt to different oxygen tensions in the BM, thus ensuring survival and correct development. A very interesting point is that these adaptations in the BM due to loss of HIF1α appear to impact on B1 B cells and autoimmunity [57]. In accordance, recent elegant experiments have revealed that HIF1α is important for expansion of CD1d^high^CD5^+^ B cells via glycolysis and production of anti-inflammatory IL-10 by those B cells [58]. Further studies performed under hypoxic conditions are required to fully explore the physiological role of glycolysis and OxPhos in pro-B and pre-B cells. Another important experimental step was to establish a system that physiologically represents human early B-cell development [59]. Studying metabolic changes during early human B cell development in vitro could provide crucial evidence about the development of autoreactivity, malignant transformation and B cell repopulation following eradication of the bone marrow (e.g., by radiation or chemotherapy) [59]. 

## 3. Signalling Pathways Linking Membrane Receptor Signals to Glycolysis and Oxidative Phosphorylation in Pro- and Early Pre-B Cells

The genes that control mitochondria and glycolysis are targets of both pre-BCR and BCR [48,60]. This indicates that the metabolic machinery of B cells integrates signals down-stream of (pre-) BCRs and growth factors [47,48], thereby, connecting the μHC idiotype with metabolism. The IL-7 and pre-BCR signalling network in pro- and pre-B cells has been reviewed in detail elsewhere [13,34]. Briefly, pro-B cells receive signals related to survival and proliferation via IL-7, Janus kinase (JAK) and signal transducer of activation and transcription (STAT) factors that enforce B cell identity via the expression of Pax5 and Ebf1. Interestingly, the B cell identity-related TFs Pax5 and IKZF1 limit glucose uptake in normal B cells and thereby support a metabolic program that leads to metabolic exhaustion when an oncogene, such as Bcr-Abl, is activated [61]. IL-7 activates the phosphatidyl-inositol-3-kinase (PI3K) pathway, leading to the activation of extracellular regulated kinase (Erk) [62] and Akt and the inactivation of Foxo1 [15,63,64]. The early pre-BCR signal also engages the PI3K cascade via Syk. In particular, the PI3K and Erk pathways control proliferation during pre-B cell development [46,47,62]. It appears, however, that PI3K activity needs to be limited by PTEN, which controls IL-7R expression, to allow pro-B cell development [22]. In mammals, mTOR is downstream of PI3K signalling in B cells (reviewed in [39]). mTOR is a kinase complex that supports IL-7-induced anabolism via glycolysis and Myc [22] (Figure 2A). The mTORC1 complex is positively regulated by Raptor, whereas the mTORC2 complex is controlled by Rictor. Anabolic mTORC1 activity is counterbalanced by AMPK (reviewed in [39]). Because Fnip1 forms a complex with AMPK and a lack of Fnip1 leads to the hyperactivation of mTOR, Fnip1 mediates the inhibitory effects of AMPK on mTOR [41,65] (Figure 2A). The conditional deletion of Raptor in B cells in mb1-Cre mice [66] led to the B cell-specific inactivation of the mTORC1 complex [67]. 

mTORC1-deficient pro- and perhaps early pre-B cells (Hardy fractions C and C’) developed in these mice, small pre-B cells (fraction D) were strongly reduced and later stages were absent. Although VDJ and VJ recombination were normal in the remaining C/C’ and D fraction cells, the expression of the IgM (μ) heavy chain was severely impaired, even when expressed as anti-hen egg lysozyme (HEL) transgenic BCR. Hence, B cell development was not rescued by the expression of anti-HEL BCR. It is unclear why mTORC1 activity is required for IgM expression. It is possible that mTORC1 controls IgM stability downstream of glycolysis and OxPhos, or it may support glycosylation via the hexosamine biosynthetic pathway and thereby stabilize the membrane expression of μHC within the pre-BCR complex (Figure 2A).

## 4. Swiprosin-2/EFhd1 as a Regulator of Glycolysis in Pro-B Cells

**Figure 3:**
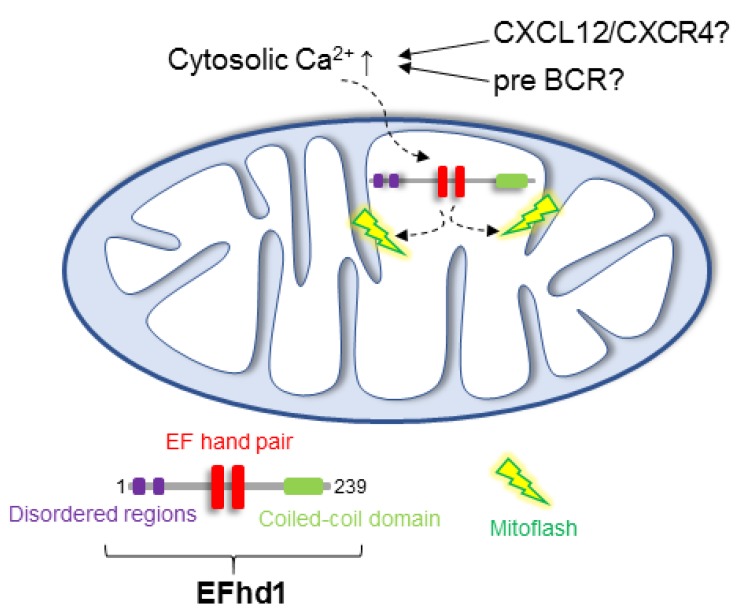
Putative mechanism for EFhd1-controlled mitoflashes in early B cells. Mitoflashes (also MitopHlashes) are bioenergetic responses to stochastic drops in the mitochondrial membrane potential (Δψ_m_). Their origin is unclear but research performed using pH-sensitive probes showed that flashes represent matrix alkalinization transients and are therefore linked to pH. It has been proposed that mitoflashes control mitochondrial metabolism and signalling in both healthy and disease states and can be triggered by increased mitochondrial Ca^2+^ concentrations. EFhd1 has been shown to mediate mitoflash activity in response to increases in mitochondrial Ca^2+^ concentrations via its two EF hands. The existence and, moreover, the consequences of this event in pro-B cells, which express EFhd1, is currently unclear. EFhd1 might translate a CXCL12 or pre-BCR induced increase in Ca^2+^ into a mitoflash, thereby coupling Ca^2+^ to mitochondrial pH regulation and proton motive force.

Swiprosin-2/EFhd1 (EFhd1) is a Ca^2+^-binding protein that localizes to the IMM and consists of an *N*-terminal disordered region, two central Ca^2+^-binding EF-hands and a C-terminal coiled-coil domain [68,69,70,71] (Figure 3). EFhd1 is a target gene of the TFs involved in B cell identity and controlling early B cell development. These TFs include Foxo1, Brg1 and Ebf1 in pro-B cells [72,73]. PTEN also promotes *efhd1* expression [44] (Figure 2A). EFhd1 becomes upregulated together with PGC-1α, uncoupling protein (UCP) 2 and other proteins involved in mitochondrial functions in the distal convoluted tubule cells of the kidney by inducing the deletion of the cytosolic Ca^2+^ buffer parvalbumin [74]. We showed that EFhd1 is expressed in primary mouse pro-B cells at the RNA and protein levels [21]. Surface expression of the pre-BCR resulted in the downregulation of EFhd1 in pro-B cells. Hence, IgM-positive B cells no longer express EFhd1 [21]. These data indicate that very early pre-B cells still express EFhd1 protein, although the half-life of EFhd1 is unknown (Figure 2A). The mechanism underlying the surface pre-BCR-mediated repression of EFhd1 is also unknown but it may involve tonic pre-BCR/BCR signals. Pro-B cells only tolerate a moderate amount of EFhd1 and pre-B cells need to downregulate EFhd1, probably to maintain mitochondrial ATP production during the pro- to pre-B cell transition [21]. 

As optimal pre-BCR signalling strength depends on efficient pairing of the newly generated μHC with VpreB and λ5, the downregulation of EFhd1 by the pre-BCR might link μHC signalling competence (the μHC repertoire) to metabolic fitness in pre-B cells. Along these lines, the Crispr-Cas9-mediated knock-out of EFhd1 as well as the its shRNA-mediated knock-down in the transformed pro-B cell line 38B9 resulted in increased glycolysis and a higher glycolytic rate and glycolytic spare capacity [21] (Figure 2A,B). We propose that the downregulation of EFhd1 in pro-B cells by pre-BCR signals is one of the mechanisms that drives pre-B cell expansion via glycolysis. We further speculate that the pre-BCR-mediated repression of EFhd1 represents a cellular readout for optimal pre-BCR signalling strength. The upregulation of PGC-1α by EFhd1 and its negative effect on glycolysis indicate that EFhd1 is a potential catabolic factor. It is thus of interest that the *efhd1* promotor is frequently methylated in tumour biopsies in colorectal cancer patients [75]. 

## 5. Signalling Pathways Linking Membrane Receptor Signals to Glycolysis and Oxidative Phosphorylation in Late Pre-B Cells

In parallel with decreasing IL-7 responsiveness, the pre-BCR initiates the expression of SLP-65/BLNK, allowing large pre-B cells to differentiate into small pre-B cells, by inhibiting the PI3K/Akt pathway and inducing a concomitant increase in FOXO1 activity [47,76,77] (Figure 2B). In small pre-B cells, PI3K and mTORC1 activity are strongly inhibited, enabling Foxo1 to suppress cell growth and facilitate LC rearrangement. EFhd1 is downregulated and its putative catabolic, that is, limiting effect on glycolysis may be overcome by a reduction in mTORC1 activity and an increase in Foxo1 activity (speculative model shown in Figure 2B). Together with Pax5, FOXO1 transactivates Rag1/2, IRF4 and p27, thereby inducing cell cycle arrest, while LC rearrangement takes place in small pre-B cells [13,73,78]. FOXO1 is also activated by ROS and upregulates superoxide dismutase 2 (*sod2*) [15]. The increase in the FOXO1 target gene, *sod2,* observed in pre-B cells [21] is compatible with the dephosphorylation and activation of FOXO1, which occurs as a consequence of reduced Akt activity [15] and might limit ROS. Interestingly, EFhd1 has been shown to be downregulated by SOD2 [79] (Figure 2B).

## 6. Putative Existence of Mitoflashes in Early B Cells—Regulated by EFhd1? 

Careful measurement of mitochondrial pH (pH_mito_) has revealed that pH_mito_ is in dynamic equilibrium with cytosolic pH (reviewed in [36]). Similarly, the cytosolic Ca^2+^ concentration is connected to the mitochondrial Ca^2+^ concentration via connections between the ER and mitochondria (reviewed in [80]) and Ca^2+^-binding proteins in mitochondria, likely including EFhd1 (reviewed in [69]). The spontaneous pH_mito_ elevations that coincide with drops in ∆ψm can occur in single mitochondria or discrete regions of the mitochondrial network and have been termed “mitoflashes” [81,82]. Mitoflashes are modulated by, for example, metabolic state, oxidative stress, developmental stage, aging and Ca^2+^ [82]. It is currently a matter of debate as to whether mitoflashes do or do not represent superoxide bursts [83] but there appears to be some consensus that they are related to pH_mito_ [81,82]. It thus has been proposed that the term “MitopHlash” [81,84] should be used and we will not address superoxide here. In general, during a mitoflash, spontaneous drops in ∆ψm are coupled to mitochondrial matrix alkalinization, thereby preserving an intact ∆*p* and enabling ongoing ATP production [36]. Experiments performed in 293 cells have shown that EFhd1 is a mitochondrial Ca^2+^ sensor of Ca^2+^-dependent mitoflashes induced by ionomycin [85]. This function of EFhd1 depends on its EF-hands, as shown in experiments with point mutations (i.e., E116A and E152A) at critical residues [86] in each of its two EF-hands. Furthermore, EFhd1 does not induce alterations in mitochondrial Ca^2+^ handling [85]. These data raise the possibility that EFhd1 is a Ca^2+^ sensor that monitors Ca^2+^ flux from the ER to mitochondria [69] and that EFhd1 may be coupled to complex I, III or IV of the respiratory chain or H^+^ coupled Ca^2+^ transporters located in mitochondria. In fact, pH_mito_ and mitochondrial Ca^2+^ are coupled [87]. There is room for speculation as to what the function of mitoflashes in early B cells might be. It is conceivable that external signals such as CXCL12 in pro-B cells [7] or successful expression of pre-BCR could activate mitoflashes by inducing signalling cascades, resulting in increased intracellular Ca^2+^ [23,24,25,26]. Assuming that mitoflashes represent mitochondrial bioenergetic phenomena that sustain the proton motive force and ATP production, the fast changes in pH_mito_ and ∆ψm may maintain quick metabolic adaption, which is vital for the activation and differentiation processes that occur in pro- and pre-B cells. EFhd1 expression in pro- and likely very early pre-B cells may contribute to the Ca^2+^-dependent control of mitoflashes, thereby, modulating CXCL12 and pre-BCR induced metabolic survival and expansion signals (Figure 3). In the case of the pre-BCR, it would, however, do so only transiently because surface expression of the pre-BCR (see above; [21]) leads to downregulation of EFhd1 via as yet unknown mechanisms. We propose that the downregulation of EFhd1 represents a sensor for optimal pre-BCR signalling strength. Pre-B cells downregulating EFhd1 faster because they express an appropriate μHC idiotype on the cell surface may switch to glycolysis faster and this may provide a competitive advantage. Hence, EFhd1 might integrate mitochondrial metabolism, glycolysis and µHC selection in BM. We envision an intimate interplay among pre-BCR-controlled genes involved in metabolism and Ca^2+^ sensing mechanisms, such as *efhd1*. This may be important for the fitness of early B cells. 

## 7. Conclusions and Perspectives

The metabolic regulation of pro- and pre-B cell development has important consequences for the expansion of normal and malignant pre-B cell clones. Under healthy conditions, it affects the normal BCR repertoire and contributes directly to adaptive immunity. To understand how the growth of normal or transformed pro- and pre-B cells is regulated, we have reviewed recent data on the regulation of glycolysis and oxidative phosphorylation in early B cells. It has become clear that pre-BCR ultimately induces metabolic quiescence because it leads to a reduction in glycolysis in late pre-B cells. The current data also suggest that large pre-B cells utilize glycolysis to sustain ATP but previous extracellular flux analyses of primary pro- and pre-B cells have missed large pre-B cells for technical reasons. Large pre-B cells may in the future be enriched from Irf4/8 double or BLNK/SLP-65 knock-out mice to clarify this issue. It remains unclear whether large pre-B cells (a) perform more glycolysis than is found in pro-B cells; (b) depend solely on glycolysis for their growth or (c) require pyruvate generated by glycolysis for ATP production via OxPhos. Hence, it remains completely unknown whether pro-B cells and pre-B cells depend in vivo on the mitochondrial respiratory chain. It will also be important to study metabolism in early B cells under hypoxic conditions, which occur naturally in BM. It is clear that HIF1α plays a role in early B cell development by promoting glycolysis in B cell progenitors. Here, we introduce the idea that recently described bioenergetic events in mitochondria (“mitoflashes”) that have been shown to maintain the proton motive force required to generate mitochondrial ATP may also occur in pro- and early pre-B cells. A Ca^2+^-binding protein that localizes on the IMM, Swiprosin-2/EFhd1 (EFhd1), might be involved in this phenomenon because it is expressed in pro-B cells and controls Ca^2+^-dependent mitoflashes. We have shown that EFhd1 becomes downregulated by cell surface expression of pre-BCR and that EFhd1 limits glycolysis in pro-B cells. We propose that EFhd1 might integrate Ca^2+^ signals with gene regulation and metabolic activity in pro- and early B cells and EFhd1 may thereby serve as a sensor for optimal pre-BCR signalling strength. The interplay between pre-BCR signalling and metabolism was clearly revealed in mice lacking mTORC1 activity in B cells. mTORC1 is required to maintain the stability of the IgM heavy chain and it therefore controls pre-BCR signalling at this level. The mechanism behind this function remains unknown but it may involve the generation of anaplerotic intermediate factors produced during glycolysis. In summary, we suggest that more extensively examining the regulation of metabolism in pro- and pre-B cells would increase our understanding of growth control and the generation of a healthy BCR repertoire. 

## Abbreviations

ALL	Acute lymphoblastic leukaemia
AMP	Adenosine monophosphate
ADP	Adenosine diphosphate
ATP	Adenosine triphosphate
6-NBDG	6-(*N*-(7-nitrobenz-2-oxa-1, 3-diazol-4-yl)amino)-2-deoxyglucose
BLP	B cell-biased lymphoid progenitor
BCR	B cell receptor
BM	Bone marrow
CAR cell	CXCL12-abundant reticular cell
CLP	Common lymphoid progenitor
ECAR	Extracellular acidification rate
EFhd1	Swiprosin-2/EFhd1
EFhd1*tg*	EFhd1 transgenic
ETC	Electron transport chain
Erk	Extracellular signal regulated kinase
FA	Fatty acid
Flt	Fms-like tyrosine kinase
Fnip1	Folliculin-interacting protein 1
HC	Heavy chain
HSC	Hematopoietic stem cell
Ig	Immunoglobulin
IMM	Inner mitochondrial membrane
JAK	Janus kinase
Ki	Knock-in
LC	Light chain
LPS	Lipopolysaccharide
∆ψm	Mitochondrial membrane potential
OCR	Oxygen consumption rate
OxPhos	Oxidative phosphorylation
Pi	Inorganic phosphate
PI3K	Phosphatidyl-inositol-3-kinase
∆pH	Proton gradient
PTEN	Phosphatase and Tensin homologue
ROS	Reactive oxygen species
SCF	Stem cell factor
SDF-1	Stromal cell derived factor
STAT	Signal transducer of activation and transcription
TCA	Tricarbon cycle
TF	Transcription factor
WT	Wildtype
